# “A uma família desconhecida”: cartas de deslocados europeus à Cruz Vermelha Brasileira, filial Rio Grande do Sul, no pós-Segunda Guerra Mundial

**DOI:** 10.1590/S0104-59702025000100006

**Published:** 2025-03-31

**Authors:** Rosane Marcia Neumann

**Affiliations:** i Professora, Programa de Pós-graduação em Educação/Universidade do Planalto Catarinense; Professora visitante, Programa de Pós-graduação em Ensino de História/ Universidade Federal do Rio Grande. Lages – SC – Brasil. rosaneneumann@gmail.com

**Keywords:** Deslocados de guerra, Cruz Vermelha, pós-Segunda Guerra Mundial, Rio Grande do Sul, War displaced persons, Red Cross, post-Second World War, Rio Grande do Sul

## Abstract

O estudo aborda as múltiplas demandas encaminhadas via cartas à Cruz Vermelha Internacional, filial de Porto Alegre, no Rio Grande do Sul, por deslocados do Leste europeu para o território da Alemanha, no período pós-Segunda Guerra Mundial (1946-1949). Entende-se essa escrita epistolar ou cartas-pedido como narrativas fragmentárias de trajetórias de vida. Objetiva-se compreender como o remetente/emissor articula os pertencimentos étnicos, religiosos e a compaixão humana para sensibilizar o seu potencial leitor rio-grandense, no intuito de receber um pacote de mantimentos, roupas, entre outras doações. Essa escrita de si descortina alguns aspectos do cotidiano miserável do pós-guerra, seus dramas e traumas, vivido e narrado por protagonistas anônimos, e o trabalho de mediação, escuta e assistência das organizações humanitárias transnacionais, como a Cruz Vermelha.

## Abstract (en)

The study addresses the multiple demands forwarded via letters to the International Red Cross, Porto Alegre branch (Rio Grande do Sul state), by persons from Eastern Europe displaced to the German territory, in the immediate post-Second World War period (1946-1949). This epistolary writing or letter-writing is understood as fragmentary narratives of life trajectories. The aim is to understand how the sender articulates ethnic and religious belonging and human compassion to sensitize the potential reader in Rio Grande do Sul, in order to receive a package of groceries, clothes, and other donations. Therefore, this writing of the self unveils some aspects of the miserable post-war daily life, its dramas and traumas, lived and narrated by anonymous protagonists, and the work of mediation, listening and assistance of transnational humanitarian organizations, such as the Red Cross.

## Migrantes em movimento

Ondas migratórias se movem na Europa: no século XIX, fruto da Revolução Industrial; na primeira metade do século XX, consequência das duas Guerras Mundiais; nas primeiras décadas do século XXI, resultado de conflitos armados externos; e, no tempo presente, da guerra interna envolvendo Ucrânia e Rússia e do conflito israelense na Faixa de Gaza.

Em diferentes momentos históricos, os fatores propulsores do deslocamento, locais de origem e de destino, alteraram-se; todavia, sua essência permaneceu inalterada: pessoas em movimento, abandonando apressadamente seus bens, seu lar e sua pátria, rumo ao desconhecido, empurrados por crises econômicas, guerras, inseguranças políticas, religiosas, climáticas etc. O pós-Segunda Guerra Mundial, até agora, produziu o maior número de deslocados de guerra na Europa – nesse contexto, consolidou-se a designação “refugiados” –, provenientes do Leste europeu. Tal deslocamento de pessoas, somado à criação de novos Estados-nação, colocou em contato e sob o mesmo território grupos étnicos e nacionalidades distintos, cujos pertencimento e identidade de grupo, construídos artificialmente – a invenção de tradições –, emergem e desmoronam, como nos conflitos em curso.

Um aspecto a notar é a seleção étnica e racial dos deslocados/refugiados, muitas vezes mascarada pelos Estados-nação. Constroem-se social e discursivamente categorias de refugiados/imigrantes visíveis e desejáveis, e refugiados/imigrantes invisíveis/indesejáveis, ou seja, um racismo seletivo, dando origem a um contingente populacional desterrado e errante. Referindo-se ao conflito entre Rússia e Ucrânia, sob a manchete “Polônia acolhe ucranianos com solidariedade sem limites”, a jornalista Magdalena Gwozdz-Pallokat (4 mar. 2022) problematiza esse acolhimento, já no enunciado, ao perguntar: “Depois de ter rechaçado refugiados de Afeganistão, Iraque e Síria, e até erguer cerca na fronteira com Belarus, poloneses abrem as portas para os que fogem da guerra de Putin. Por que comportamentos tão diferentes?”. Ela lembra que, desde o início de 2022, houve a construção de uma cerca na fronteira entre Polônia e Belarus, na tentativa de impedir a entrada de imigrantes, embora cidadãos e organizações assistenciais tenham ajudado, dentro do possível, os imigrantes. Segundo Andrzej Rychard, sociólogo da Universidade de Varsóvia, “do ponto de vista moral, a presente situação é inequívoca: não se trata da manipulação de um autocrata que deposita na fronteira polonesa os indivíduos que querem ir para o Ocidente, mas sim do ataque de um Estado a outro” (Gwozdz-Pallokat, 4 mar. 2022). Przemysław Sadura, pesquisador da mesma universidade, que investiga “os efeitos da crise migratória na divisa belarussa sobre os habitantes da área e os funcionários da fronteira polonesa” (Gwozdz-Pallokat, 4 mar. 2022) trata da mesma temática na fronteira ucraniana, notando que

essas duas crises se espelham. O que chama a atenção, entretanto, são as diferenças em sua percepção. Atualmente, todos estão engajados e ajudam os refugiados. É como um carnaval de solidariedade, um reflexo. Mas será que vai durar, quando vemos que se trata de milhões que não têm mais casa para onde voltar, e que vão permanecer na Polônia? (Gwozdz-Pallokat, 4 mar. 2022).

A solidariedade europeia do início de março de 2022 apresentou esgotamento na recepção de refugiados já no final do mês, bem como emergiram questionamentos em relação a essa política pública seletiva. Conforme o Alto-Comissariado das Nações Unidas para os Refugiados (Acnur), “em um mês, mais de 10 milhões de pessoas foram obrigadas a fugir para salvar suas vidas, deixando suas casas e pertences para trás. Mais de 6,5 milhões de pessoas estão deslocadas dentro da Ucrânia e 3,7 milhões de pessoas foram forçadas a fugir do país” (Billing, 25 mar. 2022). A maior parte dos refugiados ucranianos, mais de 2,2 milhões, foi para a Polônia, elevando ao limite máximo sua capacidade de recepção. Há ainda considerável número de russos na mesma condição, muitos buscando refúgio na Turquia – o país com maior número de refugiados no mundo, com 3,7 milhões de pessoas.

A guerra em curso, sem previsão de cessar-fogo, tem aumentado o número de baixas, deslocados internos e refugiados. Conforme os dados da Acnur (Ukraine Refugee..., 11 abr. 2023), cerca de 21 milhões de ucranianos deixaram seu país; destes, 8.167.986 refugiados estão registrados em países de toda a Europa; outros 5.038.365 estão registrados para proteção temporária ou esquemas de proteção nacional semelhantes na Europa; e mais cerca de cinco milhões e quatrocentos mil são deslocados internos. Soma-se a emigração da própria Rússia, cuja fuga em massa e sem visto direciona-se a países próximos como Cazaquistão, Quirguistão, Geórgia, Turquia, Armênia, Estônia e Letônia, cujas estimativas projetam de quinhentos mil a um milhão de russos (Ukraine Refugee..., 1 nov. 2022).

Desde o princípio da guerra, houve questionamentos de autoridades, políticos e partidos em relação à receptividade concedida aos ucranianos – e negada, por exemplo, aos sírios – em países como Dinamarca, Polônia e Reino Unido. A declaração da primeira-ministra da Dinamarca, Mette Frederiksen, em um debate no Parlamento, sintetiza a percepção europeia em relação ao refúgio: “Ser refugiado é temporário, então você tem que voltar e ajudar a construir sua terra natal quando tiver a oportunidade. Isso nos dá a oportunidade de ajudar outros refugiados” (Líderes europeus..., 25 mar. 2022).

No acolhimento e/ou rechaço do refugiado/imigrante, sobressai a percepção de Abdelmalek Sayad (1998) do imigrante como provisório, ou seja, uma força de trabalho que tem razão de existir enquanto tal. Já o refúgio, o asilo ou o deslocamento forçado são pensados como temporários tanto pelos indivíduos quanto pelo Estado, mas, em geral, perduram por longo período – a terminologia russa atual fala em “relocação” (*relokatsia*), enfatizando a natureza emergencial e temporária da fuga. Em termos jurídicos, há diferenças entre imigrantes, refugiados e deslocados; todavia, no cotidiano da sociedade de recepção ou asilo o que prevalece é a categoria do “estranho que bate à nossa porta”, como escreve Zigmunt Bauman (2017, p.9).

Refugiados da bestialidade das guerras, dos despotismos e da brutalidade de uma existência vazia e sem perspectivas têm batido à porta de outras pessoas desde o início dos tempos modernos. Para quem está por trás dessas portas, eles sempre foram – como o são agora – estranhos. Estranhos tendem a causar ansiedade por serem ‘diferentes’ – e, assim, assustadoramente imprevisíveis, ao contrário das pessoas com as quais interagimos todos os dias e das quais acreditamos saber o que esperar. Pelo que conhecemos, o influxo maciço de estranhos pode ser o responsável pela destruição das coisas que apreciávamos, e sua intenção é desfigurar ou abolir nosso modo de vida confortavelmente convencional. ... Sobre os estranhos, porém, sabemos muito pouco para sermos capazes de interpretar seus artifícios e compor nossas respostas adequadas – adivinhar quais possam ser suas intenções e o que farão em seguida. E a ignorância quanto a como proceder, como enfrentar uma situação que não produzimos nem controlamos, é uma importante causa de ansiedade e medo.

Os refugiados ou deslocados, entretanto, excluídos dessa recepção em situações como a atual, vão recorrer a quem em busca de ajuda? Quais canais de assistência e auxílio social estão disponíveis para eles? O contexto atual remete ao pós-Segunda Guerra Mundial, embora sejam situações distintas no tempo, mas próximas no espaço, pois mais uma vez envolvem as populações do Leste europeu, bem como sua sobreposição de frágeis fronteiras étnicas e nacionais, religiosas e territoriais. De acordo com Maria do Rosário R. Salles et al. (2013), em 31 de agosto de 1947, de um total de 638.759 deslocados, 328.180 encontravam-se na zona americana, 32.434 na zona francesa e 176.049 na zona britânica. Quanto à nacionalidade, 30% eram poloneses, 20% israelitas, 17% baltas (oriundos de Lituânia, Letônia e Estônia), e os demais ucranianos, russos, iugoslavos e apátridas. Para gerir essa situação, em 1946, a Organização das Nações Unidas (ONU) criou a Organização Internacional de Refugiados (OIR), com sede em Genebra, substituída em 1952 pelo Acnur.

Para além da atuação de entidades locais – ligadas ou não a governos –, os refugiados/deslocados buscavam sobreviver de outras formas, em especial, contatar familiares ou conhecidos que viviam no exterior que, em tese, se encontravam em uma situação financeira melhor e poderiam ajudá-los de alguma forma, principalmente encaminhando alimentos. Porém, nem todas as vítimas da guerra tinham esse recurso (ajuda de conhecidos no exterior), o que fez com que procurassem outros meios, como encaminhar cartas para empresas, igrejas e organizações humanitárias, que enviavam mantimentos para a Europa. Entre as organizações, a Cruz Vermelha Brasileira, filial do Rio Grande do Sul, com sede em Porto Alegre, recebeu inúmeros pedidos de ajuda por meio de cartas privadas, datadas na sua maioria entre 1945 e 1949 e, de forma mais esporádica, no decorrer da década de 1950.

A partir desse cenário, objetiva-se analisar qualitativamente um conjunto de cartas remetidas à Cruz Vermelha, filial de Porto Alegre, arquivadas na sede da organização. A leitura em escala reduzida desses fragmentos de trajetórias de vida, inscritos nas cartas, permitem o acesso ao particular, ao universo multifacetado do privado, matizando as narrativas oficiais e genéricas dos vencedores e vencidos. As cartas e seus autores “funcionam como narradores sucateiros (*Lumpensammler*), que não têm por objetivo recolher grandes feitos, mas aquilo que a sociedade ignora, nesse caso, sua própria existência” (Gagnebin, 2004, p.88-89). Do mesmo modo, o historiador também é um *lumpensammler*, e, como tal, deve recolher tudo aquilo que é deixado de lado como algo que não tem significação, algo que parece não ter nem importância nem sentido.

Na sequência, situa-se a Cruz Vermelha Brasileira, filial Porto Alegre, e o seu trabalho voltado à assistência das vítimas da guerra e, por fim, analisam-se as cartas recebidas pela organização de deslocados de guerra europeus no recorte temporal de 1945 a 1949.

### O Comitê Internacional da Cruz Vermelha e a ação humanitária

Em paralelo às organizações ligadas a governos nacionais, atuaram no contexto do pós-Segunda Guerra Mundial as organizações humanitárias independentes – juridicamente, mas não financeiramente. Um exemplo é o Comitê Internacional da Cruz Vermelha (CICV), que mantém sedes na maioria dos países e uma rede de agentes e informações bem estruturada e ágil. O Comitê Internacional para Ajuda aos Militares Feridos foi fundado em 1863, por Jean Henri Dunant, com sede em Genebra, Suíça. A organização foi consolidada com a assinatura de tratados internacionais na Convenção de Genebra de 1864. Em 1876, o comitê adotou o nome Comitê Internacional da Cruz Vermelha, que é sua designação oficial até o presente (Di Liscia, Alvarez, 2019). Trata-se de

uma organização imparcial, neutra e independente, cuja missão exclusivamente humanitária é proteger a vida e a dignidade das vítimas de conflitos armados e outras situações de violência, assim como prestar-lhes assistência.

O CICV também se esforça para evitar o sofrimento por meio da promoção e do fortalecimento do direito e dos princípios humanitários universais (CICV, s.d.).

Como princípios, o CICV defende a humanidade, a imparcialidade, a neutralidade, a independência, o voluntariado, a unidade e a universalidade. Trata-se de “uma instituição mundial, na qual todas as Sociedades têm iguais direitos e dividem iguais responsabilidades e deveres, ajudando-se mutuamente” (CICV, s.d.). O “colete com a cruz vermelha” é um salvo conduto e “à prova de balas” para adentrar e socorrer as populações em territórios em guerra, desastres, emergências ambientais, epidemias, dentre outras ações humanitárias.^1^ A ramificação do CICV nos países se efetiva via formação das sociedades nacionais da Cruz Vermelha. Atualmente, a organização tem cerca de 12 mil funcionários em oitenta países; é financiada, sobretudo, por doações voluntárias dos governos e das Sociedades Nacionais da Cruz Vermelha e do Crescente Vermelho.

A Cruz Vermelha Brasileira foi criada em dezembro de 1908, com sede no Rio de Janeiro, então capital do país. Teve como primeiro presidente o médico sanitarista Oswaldo Cruz. Apresenta-se como sociedade de socorro voluntário, autônoma, recebendo recursos do poder público, de instituições e indivíduos. O registro e o reconhecimento da entidade nos âmbitos nacional e internacional se deram entre 1910 e 1912. A Primeira Guerra Mundial (1914-1918) foi fator de impulso para sua consolidação no país, com filiais espalhadas em todo o território nacional – hoje, são 18 unidades. Em 1940, foi criada a filial do estado do Rio Grande do Sul, com sede em Porto Alegre (História, s.d.).^2^

Nos tratados internacionais do pós-Segunda Guerra Mundial, o Brasil foi um dos países signatários para acolher imigrantes “deslocados”. Segundo os dados do Departamento de Imigração do Ministério do Trabalho, Indústria e Comércio, o país recebeu 22.009 imigrantes “deslocados”, dos quais 11.079 (cerca de 51%) dirigiram-se a São Paulo; 4.606 (quase 21%) ao Paraná; 2.160 (8,8%) ao Rio Grande do Sul; 1.705 (7,7%) ao Distrito Federal; 852 (3,8%) a Goiás; 760 (3,4%) a Santa Catarina; 553 (2,5%) ao Rio de Janeiro; 463 (2,1%) a Minas Gerais; 386 (1,7%) à Bahia. Os demais estados receberam números ínfimos de imigrantes. Segundo dados da *Revista de Imigração e Colonização* de 1950 (citada em Salles, 2013), São Paulo se sobressai em função da oferta de empregos urbanos e da possibilidade de absorção de mão de obra qualificada.

No contexto europeu de pobreza e miséria decorrente da Segunda Guerra Mundial, deslocados de guerra em seu território e refugiados, desamparados pelo Estado, recorreram ao escritório ou campo de operação da Cruz Vermelha mais próximo, em busca de ajuda humanitária. As frentes de atuação da Cruz Vermelha foram múltiplas: assistência médica aos feridos na guerra,^3^ coleta e envio de mantimentos, busca por desaparecidos, ajuda para emigração, entre outras. O envio de mantimentos foi, durante o período de 1945 a 1949, a maior frente de atuação da filial do Rio Grande do Sul. Em diversas cartas-pedido [*Bittbriefe*], eram solicitados “pacotes de amor” [*Liebesgabenpakete*], os quais seguiam um padrão.

Os pacotes devem ter o peso de 5kg, com invólucro branco (de pano) no qual deve escrever o endereço a tinta e bem legível, assim como o nome do remetente. Quanto ao que deve remeter, será: de 1 a 2kg de café, banha, açúcar, arroz, sabão, chá, chocolate, cereais, conservas enlatadas, féculas alimentícias, todas as espécies de alimentos, com exceção de: azeite, banha de coco, fumo e artigos de borracha. Devo dizer que cada um destes alimentos não deve exceder a 1kg. Além disso, poderá enviar também fazendas, roupas novas e usadas, calçados, couro etc. Peço o obséquio de remeter os pacotes em domicílio. A taxa é de Cr$ 50,00 por cada pacote de 5kg. Ficará ao critério de V.S. remeter um ou mais pacotes ao mesmo endereço (Müller, 26 jun. 1948).

Nota-se que a Cruz Vermelha teve um papel importante como mediadora entre os demandantes por auxílio e os possíveis benfeitores, articulando uma rede internacional de ajuda humanitária. Um registro dessa atuação e suas ramificações são as cartas enviadas por refugiados e deslocados à filial da Cruz Vermelha no Rio Grande do Sul, em Porto Alegre, no período em questão.

### Cartas: uma fresta na janela

Em meados do século XX, o suporte e meio utilizado pelos deslocados de guerra em seu próprio território e os refugiados foi a escrita de cartas, nas quais expressavam suas demandas materiais urgentes e, para justificar seu pedido, narravam sua tragédia, fornecendo ao seu leitor um fragmento de sua história de vida, na expectativa de que essas cartas chegassem às mãos de alguém, sensibilizando-o.^4^ Simultaneamente, essas cartas desempenharam o papel de testemunha e denúncia das condições materiais, sociais e sanitárias dessas populações marginalizadas pela guerra, não contempladas pelas políticas públicas dos Estados.^5^ Entre os signatários dessa escrita epistolar estava o CICV, e uma parcela das cartas tinha como destinatária a Cruz Vermelha Brasileira, filial do Rio Grande do Sul.

A correspondência, como gênero epistolar, incide no âmbito da vida privada do autor e do leitor, pois é um documento escrito dentro de uma relação entre indivíduos, e o nível confidencial modela a sua linguagem (Granda, 2016). Para Michel Foucault (1992, p.6), “a carta enviada atua, em virtude do próprio gesto da escrita, sobre aquele que a envia, assim como atua, pela leitura e a releitura, sobre aquele que a recebe”. A carta é quase um face a face entre emissor e receptor, colocando-os frente a frente. Escrever uma carta, entendida como um estilo epistolar,

é ‘mostrar-se’, dar-se a ver, fazer aparecer o rosto próprio junto ao outro. E deve-se entender por tal que a carta é simultaneamente um olhar que se volve para o destinatário (por meio da missiva que recebe, ele sente-se olhado) e uma maneira de o remetente se oferecer ao seu olhar pelo que de si mesmo lhe diz. De certo modo, a carta proporciona um face a face (Foucault, 1992, p.8).

As cartas são, justamente, o testemunho desse esforço, dessa tarefa (quase) impossível: a busca constante para reconstruir, ou manter inalterado, por meio da escrita, aquilo que a emigração havia irremediavelmente interrompido ou modificado (Croci, 2008, p.17). A carta, como fruto desse esforço de superar as distâncias, possibilita compreender fragmentos do cotidiano desse sujeito, as transformações sociais, culturais e identitárias. Todavia, nem todos os deslocados/refugiados escrevem, por razões diversas. As cartas são produtos do íntimo, do doméstico, do privado, uma troca de informações entre indivíduos, mas também são públicas e coletivas, visto que muitas vezes são lidas ou escritas por terceiros, são transcritas ou datilografadas, traduzidas, manipuladas ou censuradas. Além disso, muitos imigrantes, devido à falta de recursos, buscavam entidades públicas ou privadas para encaminhar suas cartas.

As cartas arquivadas na sede da Cruz Vermelha estadual, em Porto Alegre, partem do domínio privado e familiar e destinam-se ao âmbito público de uma organização de ajuda humanitária. Os deslocados de guerra e refugiados, diante de um horizonte nebuloso, escreveram sua carta-pedido ou carta-relato de forma “indeliberada” – isto é, sem preocupação estilística, deliberações ou reflexões, de forma espontânea e informativa.^6^ Nessa perspectiva, nas cartas em questão, os autores não se delongam em narrar as suas experiências de vida – talvez inarráveis, como percebe Walter Benjamin (1987b) em relação aos sobreviventes no pós-Primeira Guerra Mundial, os quais emudecerem, pois o que presenciaram e viveram era impossível de ser narrado em palavras.

Esses homens e mulheres, ao escrever uma carta de próprio punho, tinham como objetivo primeiro descrever sua condição do tempo presente de carestia, miséria, doença, numa tentativa desesperada de sobreviver no aqui e agora. *A posteriori*, essas escritas configuram-se como rastros ou vestígios de sua existência, tirando-os do anonimato e esquecimento. Todavia, cabe lembrar que a escrita epistolar está impregnada pelos filtros usados pelo seu remetente e, muitas vezes, pela censura prévia de instâncias hierárquicas difusas. Há cartas, por exemplo, que exibem em anexo uma “carta de recomendação” ou *Bescheinigung*, assinada por uma autoridade local – prefeito, chefe do distrito, pastor, padre. É o caso da carta de Liesbeth Kahnert, residente em Untereuerheim (município de Grettstadt, na Baixa Francônia, no distrito de Schweinfurt), na Zona de Ocupação Americana, escrita em 17 de abril de 1948. Ela residia com a filha Regina, de 4 anos, e o marido Reinhold Kahnert, retornado há poucas semanas de um cativeiro francês. Em sua missiva, Liesbeth dá conta de sua fuga de Marwald, uma vila da Polônia, localizada na Voivodia da Vármia-Masúria, no distrito de Ostróda, na comuna de Dabrówno. O certificado anexo ou “carta de recomendação” informava que “o Sr. Reinhold Kahnert, Untereuerheim Landhaus Söllner, distrito de Schweinfurt/Ufr. é um refugiado do distrito de Marwalde em Osterode/Prússia Oriental. O conjunto familiar é composto por 3 pessoas” (Kahnert, 17 abr. 1948). Logo, deduz-se que a carta passou do domínio privado ao domínio público, no caso, a autoridade local, bem como o documento anexo atestava a veracidade das informações fornecidas, no que se refere à condição de refugiado e à composição do núcleo familiar.

Em termos de estrutura, as cartas variam quanto a enfoque de conteúdo, densidade narrativa e extensão, bem como a elementos anexos, compostos por atestados de boa conduta, recomendações, certidões, fotografias. Infere-se que o remetente, no intuito de comprovar a veracidade de sua narrativa e de sua demanda, buscou o aval das autoridades locais – o que abre brechas para acreditar em um universo paralelo de cartas, cuja veracidade das informações e da narrativa eram duvidosas. Todavia, “devemos insistir na sinceridade desse tipo de texto, pois geralmente é escrito com um propósito prático e em uma atmosfera emocional que faz com que eles transmitam sentimentos e emoções insubstituíveis na vida dos protagonistas e, portanto, é difícil distorcê-los ou falsificá-los” (Granda, 2016, p.11).

Sob essa perspectiva, os autores das cartas registraram e socializaram o seu testemunho de um contexto/fato datado no tempo e no espaço. “Todo testemunho é único e insubstituível. Esta singularidade absoluta condiz com a singularidade da sua mensagem. Ele anuncia algo excepcional. ... Por outro lado, o testemunho também se quer compreensível e, mesmo, o testemunho se quer exemplar” (Seligmann-Silva, 2008, p.72). Marc Bloch (2019, p.57), em texto de 1914, lembra que “as testemunhas não são todas sinceras nem suas memórias sempre fiéis, de forma que não nos é possível aceitar qualquer depoimento sem controle”. Assim, “longe de tomar a infidelidade testemunhal como um entrave, Bloch requeria o rigor da crítica histórica e erudita para controlar e assumir a dimensão testemunhal como etapa central na operação historiográfica, observado o rigor metódico”, ampliando “a compreensão sobre o passado com a formulação de perguntas ancoradas na experiência presente” (Fredrigo, Gomes, 2020, p.10, 12).

A escrita das cartas ocorre no presente do seu escrevente, no aqui e agora, ou seja, no o tempo do acontecimento – semelhante a uma fotografia que congela uma cena. Em seu registro, deixa entrever por uma fresta um pequeno fragmento de sua condição, seu cotidiano e seu “eu”, e, de imediato, elenca a sua demanda. Nesse sentido, as cartas como “escritas de si” são distintas das histórias de vida, elaboradas anos após os fatos por um processo de memória e esquecimento, ou seja, o tempo da narrativa – como, por exemplo, a história de vida de uma deslocada de guerra que emigrou ao Brasil, estudada por Roseli Boschilia (2021).

Por fim, como enfatiza Benjamin (1987b, p.205), em ensaio de 1933, o ato de narrar “é uma forma artesanal de comunicação”, na qual o narrador deixa impressa a sua marca “como a mão do oleiro na argila do vaso”, diferenciando-se, assim, de uma informação ou de um relatório. E, entre as narrativas escritas, “as melhores são as mais próximas a narrativa oral” (p.199). Ao debruçar-se na leitura das cartas dos refugiados e deslocados de guerra, há o risco de cair na armadilha de buscar explicações e perder o foco na história em si. “Metade da arte narrativa está em evitar explicações. [o leitor] é livre para interpretar a história como quiser, e com isso o episódio narrado atinge uma amplitude que não existe na informação” (p.203).

### Narrativas do inarrável: as cartas dos refugiados e deslocados de guerra

Perseguir os rastros dos deslocados de guerra e refugiados, acessar suas narrativas, perpassadas pelo trauma, pelo silêncio e pelo não dito, é tarefa complexa, com a qual se deparam os pesquisadores, tanto no presente quanto no passado, independentemente do suporte das narrativas e dos meios de comunicação. Tais narrativas implicam narrar o inarrável, seja no papel de testemunha ocular, vítima ou protagonista na linha de frente da batalha, isto é, o testemunho sobre o cotidiano do sujeito comum, atropelado pelo conflito, que, ao seu término, retorna à sua vida ordinária.

Nesse contexto, as cartas endereçadas à Cruz Vermelha, filial de Porto Alegre, no imediato pós-Segunda Guerra Mundial e preservadas em seu arquivo não impactam pela quantidade, mas pela particularidade do conteúdo, pois cada carta é uma testemunha de sua época, ao narrar uma trajetória de vida única e de seu protagonismo. Ao escrever à uma organização internacional ramificada em diversos países, esses homens e mulheres buscaram um canal de escuta, ao qual confiaram a sua história, além de uma possibilidade remota de obter auxílio material. Essas escritas de si produzidas por pessoas comuns – o que se denota pelo vocabulário usado, erros de ortografia e verbais – saíram do fluxo privado e chegaram ao espaço público de uma organização internacional, na expectativa de que fossem lidas pelos seus destinatários. Ao narrar o inarrável do seu cotidiano, além de sensibilizar o leitor em prol de sua causa, lembravam ao mundo que o conflito bélico havia acabado, mas as consequências da guerra permaneciam e recaíam diretamente sobre a população civil: desagregação familiar, pessoas mutiladas, doenças, miséria, desaparecidos e mortos.

A prática do gênero epistolar em situação de dor é intensa. A correspondência é um elemento de primeira necessidade. A angústia e os afetos contidos nas cartas são testemunhos da situação de incerteza. Em relação ao registro da intimidade e ao pudor que se pode sentir ao ler as cartas pessoais, é significativa a informação da situação comum que contêm. ... Nas cartas pessoais, normalmente, há certa simultaneidade entre o que se está fazendo e o que se está sentindo. É um testemunho no sentido que se faz o que se diz. Um dito e feito: ‘Te escrevo essas linhas para contar-te que estamos bem’. Há uma simultaneidade, uma sincronia, entre a escrita e a vivência que se relata. As lembranças são frescas, imediatas. Entretanto, não se esquecem de certos nomes, situações ou lugares; tampouco há transferências ou mitificações que naturalmente se instalarão mais tarde na memória (Iturra, 2020, p.87).

As correspondências escritas por refugiados e deslocados do seu território da Europa oriental, ocupado pela URSS, e realocados na Alemanha e países limítrofes, tratam da “história do sensível” e dos traumas. Na segunda metade do século XX, a corrente de estudos denominada *trauma studies* passou a advogar a reflexão sensível sobre as possibilidades críticas da narrativa para a representação da experiência traumática:

Fruto de variadas ponderações, esse campo, interessado pela memória traumática, concretizou-se, vinculando áreas interdisciplinarmente. Nesse sentido, a discussão em torno da narrativa e as representações traumáticas afirma uma contribuição distintiva, considerando, ainda, o bom acúmulo de pesquisas, nacional e internacionalmente (Fredrigo, Gomes, 2020, p.13).^7^

Na definição de Dominique LaCapra (2020, p.31),

o trauma é uma experiência abaladora, que distorce a memória no sentido ‘ordinário’ e pode torná-la particularmente vulnerável e falível durante a narração dos eventos. O que se tem chamado de memória traumática está ligado a sintomas da experiência traumática, a exemplo de pesadelos, *flashbacks*, reações sobressaltadas e comportamentos compulsivos.

LaCapra (2020, p.32) assevera que, no caso das narrativas traumáticas, “o testemunho e o testemunhar podem não ser confiáveis quanto ao relato preciso dos eventos, em grande medida, por conta dos efeitos desorientadores do próprio trauma”. Argumenta que há uma distinção complexa entre presenciar como testemunha, prestar testemunho e oferecer comentários de um tipo ou de outro.

O presenciar como testemunha, nesse uso, refere-se ao ato de vivenciar a experiência de um evento, o que pode resultar em diversas formas não verbais, incluindo sintomas pós-traumáticos. ...

Dar testemunho envolve a tentativa de lidar, ou fazer um relato da experiência que a pessoa teve em primeira mão, ou pela qual precisou passar. Em determinado sentido, a experiência de dar um testemunho pode ser entendida como uma tentativa falível de uma pessoa de verbalizar ou, de outra forma, articular o que ela vivenciou como testemunha. O testemunho em si é, ao mesmo tempo, ameaçado e, de certo modo, autenticado ou validado, na medida em que contém as marcas dos efeitos sintomáticos de um trauma, podendo ser até mesmo completamente consumido e distorcido por eles. Mas o testemunho pode se desdobrar na forma de diversos tipos de comentários relacionados à experiência vivida e aos eventos em seu entorno (LaCapra, 2020, p.31-32).

Ainda conforme o autor, a opção e a “capacidade de dar testemunho já é, em si, um componente da sobrevivência”, e o testemunhar implica uma relação de confiança e escuta, ou seja, a presença efetiva ou virtual do ouvinte ao qual o indivíduo conta sua história. O testemunho, para além da narrativa, pode adotar diferentes formatos, como o ensaio, o poema, a piada, a canção, a dança, a pintura, a escultura, a fotografia (LaCapra, 2020, p.32).

No presente estudo, os testemunhos se expressam por meio da escrita epistolar. Refugiados e deslocados de guerra, diante das dificuldades e da miséria, desamparados pelo Estado, buscaram canais de interlocução e escuta, como testemunhas dos escombros do pós-guerra, bem como amparo material. Nesse emaranhado de traumas e demandas, houve aqueles que optaram por narrar sua trajetória em uma carta, dirigindo-a a um órgão transnacional – a Cruz Vermelha –, elegendo-o como mediador ou destinatário final, na expectativa da missiva encontrar um destinatário, um ouvinte e até um interlocutor. São testemunhos de sujeitos comuns que, em sua maioria, não dominam a arte da narrativa, expressando de forma sintética e objetiva suas impressões e necessidades.

No rastro da guerra, emergiram fileiras de populações deslocadas, obrigadas a abandonar a sua aldeia, a sua pátria, e migrar para locais pré-definidos. Outros optaram por abandonar seu local de origem e emigrar para diversos países. Mesclam-se e surgem nesse cenário novas terminologias para definir essas populações em movimento e sua condição jurídica, nem sempre muito claras para os sujeitos envolvidos. Em 1943, enquanto a guerra se desenrolava no campo de batalha, Hannah Arendt escreveu e publicou na imprensa norte-americana o texto “We refugees”, traduzido e publicado em 2013 como livro, com o título *Nós, os refugiados*, no qual apresentou vários elementos para pensar a categoria refugiado e o que a sociedade esperava dele, sinalizando para a complexidade da condição desses sujeitos, bem como a construção de uma identificação com essa categoria.

Em primeiro lugar, não gostamos de ser chamados ‘refugiados’. Chamamo-nos uns aos outros ‘recém-chegados’ ou ‘imigrantes’. ...

Um refugiado costuma ser uma pessoa obrigada a procurar refúgio devido a algum ato cometido ou por alguma opinião política. Bom, é verdade que tivemos que procurar refúgio; mas não cometemos nenhum ato, e a maioria de nós nunca sonhou em ter qualquer opinião política radical. O sentido do termo ‘refugiado’ mudou conosco. Agora ‘refugiados’ são aqueles de nós que chegaram à infelicidade de chegar a um novo país sem meios e tiveram que ser ajudados por comitês de refugiados. Antes de esta guerra começar éramos ainda mais sensíveis a sermos chamados refugiados. Demos o nosso melhor para provar aos outros que éramos apenas imigrantes comuns. Afirmávamos que tínhamos partido pela nossa própria vontade para países da nossa escolha e negávamos que a nossa situação tivesse algo a ver com ‘supostos problemas judaicos’. Sim, erámos ‘imigrantes’ ou ‘recém-chegados’ que tínhamos deixado o nosso país porque, num belo dia, não nos convinha mais ficar, ou puramente por razões econômicas. Queríamos reconstruir as nossas vidas, isso era tudo. De modo a reconstruir a vida tem que se ser forte e otimista. Portanto, éramos bastante otimistas.

Com efeito, o nosso otimismo é admirável, mesmo que sejamos nós a dizê-lo. A história da nossa luta finalmente tornou-se conhecida. Perdemos a nossa casa, o que significa a familiaridade da vida cotidiana. Perdemos a nossa ocupação, o que significa a confiança de que tínhamos algum uso neste mundo. Perdemos a nossa língua, o que significa a naturalidade das reações, a simplicidade dos gestos, a expressão impassível dos sentimentos. Deixamos os nossos familiares nos guetos polacos e os nossos melhores amigos foram mortos em campos de concentração, o que significa a ruptura das nossas vidas privadas (Arendt, 2013, p.7).

Nas cartas, os remetentes ora se identificam como deslocados de guerra, ora como refugiados, indício da fluidez e indefinição dessas categorias. Uma permanência nas cartas é a referência à condição socioeconômica prévia ao deslocamento: de forma genérica, trata-se de sujeitos com formação escolar e profissional, detentores de uma posição no mercado de trabalho e bens móveis; porém, repentinamente, foram obrigados a abandonar tudo, deslocar-se para outra região dentro do próprio território, como deslocados, ou para outro país na categoria de refugiado, acarretando as implicações decorrentes desse movimento. Nas narrativas, sobressai a dor da perda de sua *Heimat*/aldeia, sua casa, seus pertences e bens, seus laços familiares e sociais, somada à inconformidade com a condição socioeconômica desfavorável do presente.

### Deus, pátria e família: aspectos de sensibilização das narrativas

Ler e analisar histórias comovedoras e traumáticas, sem deturpar sua essência, é um desafio para o pesquisador. Assim, optou-se por dar voz às narrativas desses homens, mulheres, jovens e idosos, deslocados e refugiados, reproduzindo-as na íntegra, traduzidas ao português – abstendo-se de falar por eles, mas a partir deles.

Conforme já exposto, a condição de deslocado/refugiado é anômala na vida desses sujeitos e impacta diretamente na sua concepção de ser humano e de seus valores éticos e morais. Partindo de seu lugar de fala, o ato de “pedir” ajuda, acionando redes familiares, sociais e assistenciais, é por si só humilhante. A decisão e o ato de escrever uma carta subjaz a percepção subjetiva de “mendicância” e, por extensão, remete a um sentimento de humilhação, ferindo o orgulho próprio de provedor de si e de sua prole.^8^ Como artifício para atenuar o ego ferido, o remetente assegurava tratar-se de pessoa com boas referências e antecedentes, empurrado à margem da sociedade pelos desdobramentos do contexto pós-guerra. “A grande necessidade me obriga a dirigir um pedido a você”, escreveu Benno Eisenheim, morador de Behringen (uma parte do município de Bispingen, no distrito de Heidekreis, na Baixa Saxônia), em 2 de fevereiro de 1948. Justificado o contexto da escrita e sem destinatário predefinido, narra a tragédia familiar:

Sou um refugiado do Leste [*Ostflüchtling*] e recentemente retornei de [um campo de] prisioneiro de guerra inglês. Minha esposa e seus cinco filhos fugiram dos russos em janeiro de 1945 e não puderam levar nada com eles. A miséria e a necessidade são grandes, tanto as crianças como nós não temos nada em nosso corpo, as crianças são subnutridas. Temos fé e esperamos que você cuide de nós (Eisenheim, 2 fev. 1948).^9^

Na ausência de vínculos familiares ou redes sociais no exterior, os deslocados/refugiados utilizam outras estratégias para sensibilizar o potencial leitor da missiva, como o seu vínculo pátrio – *Unsere Heimat ist Westpreussen* (Nossa *Heimat* é a Prússia Ocidental) –, remetendo à sua pátria de origem, da qual foram expulsos ou deslocados, e à sua presença provisória e temporária em terras estranhas. Em outros casos, na falta de um interlocutor, acionam o pertencimento religioso como elo.

Amados fiéis, queridos irmãos e irmãs no Senhor!

Quero lhe dirigir um grande pedido. Pois eu me encontro em grande miséria. Pois em 20/01/1945 tive que deixar minha propriedade [*Hof*] e minha querida pátria [*Heimat*] com meus 5 filhos pequenos durante a noite. Os russos não estavam muito longe. Meu marido era um prisioneiro de guerra na época. Mas fui detida pelos russos e perdi meus últimos bens. Eu só encontrei meu marido novamente fora do cativeiro em 1947, e ele estava miserável. Eu não sei como alimentar meus quatro filhos. Alguns morreram de fome com os russos. Os outros têm doença pulmonar (Lüdeking, 26 fev. 1948).

Pertencimentos étnico e religioso, o processo de deslocamento e a reunião familiar marcam a narrativa da carta de Hildegart Lüdeking, fixada em Bega, na Westfália, escrita em 26 de fevereiro de 1948. A miséria na qual vivia com seus quatro filhos e o marido, cujas necessidades básicas não eram atendidas, carecendo de mantimentos e vestuário, levou-a recorrer à Cruz Vermelha.

Eu recorro a vocês, meus queridos irmãos e irmãs, em busca de ajuda. Cada doação, que seja pequena, vale muito para mim. Até mesmo um pequeno presente [pacote de amor] vale muito para mim. Preciso acima de tudo de roupa e roupa de cama. Eu nasci em 08/08/1910. 37 anos de idade. Três filhos de 12, 10 e 6 anos de idade. Uma filha de 6 anos de idade.

Meu marido tem 40 anos de idade. Não tenho algodão para meias ou roupas íntimas. ... O Senhor o recompensará. Em firme confiança o saudamos calorosamente (Lüdeking, 26 fev. 1948).

Em meio aos escombros do pós-guerra, as crianças e os adolescentes eram agentes ativos na luta pela sobrevivência individual e familiar – o índice de mortalidade infantil em 1945 era de uma em cada quatro crianças (Judt, 2008). Desprovidos do mínimo, os filhos da guerra também recorreram à Cruz Vermelha, em busca de um *Liebesgaben* [*Liebes* = de amor; *Gabe* = donativo, “pacote de amor”]: é o caso de um(a) dos(as) filho(as) órfãos(ãs) de pai da família Althoff, que se encarregou da escrita de uma carta-pedido:

Prezada Comunidade Cristã! ‘Quando a necessidade é maior, a ajuda de Deus está mais próxima’. Isto se repete constantemente. Mamãe não sabe o que nos dizer, o porquê estamos carentes de tudo. ... Somos três irmãos e irmãs e vivemos com mamãe em uma comunidade familiar próxima. Nosso querido pai foi morto na Rússia em 1942. Desejamos a todos o melhor e permanecemos, com saudações calorosas (Althoff, 6 mar. 1948).

O esforço da mulher, viúva e mãe, como chefe da família Althoff, possivelmente não passava despercebido aos olhos dos filhos, bem como a sua impotência em meio à carestia. A carta, como documento e narrativa, foi escrita e assinada em nome da família, e não traz maiores indícios sobre as circunstâncias de sua produção – talvez uma escrita familiar conjunta, uma atividade escolar, ou ainda uma mãe analfabeta, que delegou a redação aos filhos; ou, ainda, uma estratégia de sensibilização, valendo-se do apelo infantojuvenil. Para além das conjecturas, o fato é que a família recorreu ao auxílio da Cruz Vermelha para obter um pacote de mantimentos.

A “família” como elemento narrativo está presente na quase totalidade das cartas do acervo da Cruz Vermelha Brasileira, filial do Rio Grande do Sul. A sensibilidade da questão lida com as sensações, com o emocional, com a subjetividade, com os valores e os sentimentos de quem lê a carta, indo diretamente da percepção individual e obedecendo outras lógicas e princípios que não os racionais (Pesavento, 2005). Portanto, escrever de uma família para outra família ocasiona uma abordagem direta, cara a cara, ou seja, sensibiliza o leitor fazendo com que reflita sobre sua própria realidade.

Prezada Família Desconhecida

‘Onde a necessidade é maior, a ajuda de Deus é a próxima’.

Neste espírito, tomamos a liberdade de nos aproximarmos de vocês com um pedido sincero no vínculo de nossa fé comum em nosso Senhor Deus. Por mais incomum que este caminho possa parecer para você, acredite-nos, é difícil para nós apresentar-lhe nosso pedido.

O que Deus faz é sempre correto e, por mais difícil que seja para todos nós, devemos carregar nossa cruz corajosamente. A guerra, que trouxe grande sofrimento a quase toda a humanidade, também atingiu duramente nossa família. A vocês, querida família desconhecida, somos uma família de cinco membros da agora área polonesa ocupada da Silésia. Perdemos tudo; nossa casa, nossos bens e nosso querido pai. Mas sabemos que os bens terrenos por si só não podem nos fazer felizes, por isso temos que lutar longe de casa nestes tempos amargamente difíceis e graves para a Alemanha. Não precisamos lhe dizer como todos nós, sem-teto [deslocados/refugiados, sem lar], estamos indo aqui. Não queremos ir mais longe e gostaríamos de apresentar brevemente nosso pedido a você, querida família desconhecida e distante.

Por favor, tenha a bondade de nos ajudar em nossa necessidade. Nós, membros da Igreja Católica, podemos esperar ajuda.

Portanto, pedimos com sinceridade que não nos esqueçam. De cinco desabrigados da Alemanha (61 anos, 31 anos, 26 anos, 21 anos e 2 anos).

Com antecedência, um sincero ‘Deus te abençoe’ (Goetz, 3 jun. 1947).

A carta de Maria Goetz (Oberlichtenau, distrito de Sachen, Zona de Ocupação Soviética), datada de 3 de junho de 1947, traz como marcas o apelo à figura da família – “uma família desconhecida” – e o apelo religioso – “nós, famílias católicas” – como laços invisíveis que conectam o emissor e o receptor da missiva, ancorados em valores fundacionais das suas sociedades em contato. É notório que, em meio a catástrofes, aumenta a predisposição das pessoas de buscar orientação e conforto nas igrejas, ou, como afirma Bessel (2010, p.303), a catástrofe leva “o povo de volta para Cristo”. Se, por um lado, a religião serve como elemento para sensibilizar seu leitor, por outro lado, traz indícios da resiliência e resignação dessas famílias desalojadas, deslocadas/refugiadas, ao carregar a sua “cruz corajosamente”, na esperança de dias melhores, restando-lhes os elementos basilares: a vida, a família e a fé.

### Heimatlosen: organização étnica

As cartas em questão, em sua maioria, narram deslocamento e fugas individuais ou familiares, permitindo espiar por uma fresta singular. Contudo, há aquelas que mencionam indícios sobre a organização dos refugiados, como uma estratégia coletiva, ampliando as possibilidades de obter ajuda humanitária de múltiplas organizações no exterior. Nesse rol, enquadra-se a carta assinada por Paul Ruff, representante do “Comitê de expulsos da comunidade [aldeia] de Wiescherhöfen”, Westfália, escrita em nome de quatrocentos alemães deslocados, encaminhada à Caritas – e possivelmente reenviada à Cruz Vermelha, filial do Rio Grande do Sul – datada de 1º de setembro de 1947.

O signatário desta carta lhes fala em nome de 400 alemães que foram expulsos de sua pátria [*Heimat*]. A necessidade extrema e miséria desses deslocados nos levou a não deixar pedra sobre pedra para tornar nossa situação desesperadora mais suportável. Uma vez que mesmo uma ajuda modesta é viável no vosso país, a decisão de pedir a vossa ajuda amadureceu – após superar todas as inibições e diante da necessidade. Nos falta tudo para suprir as necessidades mais básicas para levar uma vida que seja mesmo remotamente humana. Como resultado da mais terrível de todas as guerras, milhões de nós, alemães da fronteira, fomos expulsos de nossas casas, propriedades [*Hof*] e pátrias [*Heimat*]. Os poloneses e russos brutalizaram e desavergonhadamente nos despojaram até mesmo das roupas mais básicas, e nós estávamos à deriva rumo a um destino incerto. Depois de longa caminhada sob dificuldades e privações inimagináveis, às quais muitos de nós não sobreviveram, chegamos – física e mentalmente arruinados – à nossa atual casa, uma pequena aldeia chamada ‘Wiescherhöfen’, perto de Hamm, na Westfália (Ruff, 1 set. 1947).

Denota-se, a partir da leitura da carta, que pedir ajuda a organizações no exterior foi uma decisão tomada coletivamente, ao constatar que as organizações humanitárias em atividade no local – como a própria Cruz Vermelha alemã – não davam conta de suprir as necessidades básicas do grupo. Paul Ruff, como porta-voz de quatrocentos alemães, tinha contra si o fato de serem alemães natos, em um contexto de condenação e responsabilização internacional da Alemanha pela guerra e pelo holocausto. Organizações humanitárias internacionais, como as caridades britânicas e o grupo de escoteiros “não tinham vontade de trabalhar para ajudar [os alemães] quando nacionalidades inocentes que eles tinham tratado como lixo estavam precisando de assistência” (Shephard, 2012, p.163). Da mesma forma, organizações ligadas às Nações Unidas também restringiram o socorro aos alemães – a Administração das Nações Unidas para Assistência e Reabilitação (Unrra, na sigla em inglês) foi proibida de ajudar os alemães, e a OIR também excluiu os alemães do seu quadro de auxílios.^10^

A região do Ruhr, onde Paul Ruff e os demais alemães deslocados estavam instalados, era conhecida como o centro industrial e de mineração da Alemanha, e, como tal, foi uma das regiões mais atingidas pelos bombardeios.

Situada em uma área industrial no Ruhr, quase completamente destruída pelos efeitos da guerra, a população local, em sua maioria indigente e sem abrigo, mesmo com a melhor vontade do mundo, é incapaz de ajudar os desabrigados [*Heimatlosen*], esta é a área em que estamos alojados. A fome é nossa sórdida companheira e o espectro do inverno frio estende seus braços na nossa direção, já que poucos de nós têm roupas suficientes para se manterem aquecidos (Ruff, 1 set. 1947).

Conforme Ben Shephard (2012, p.168), essa região foi historicamente dependente, “no que diz respeito à maior parte de sua comida, do polo de produção de grãos da Alemanha, que eram as ricas áreas agrícolas do leste da Prússia, agora divididas entre Polônia e União Soviética, ou seja, estava em ruínas e não produzia alimentos”, o que fazia com que a população dependesse da atuação das organizações internacionais e humanitárias.

Por isso, temos um pedido urgente a vós: ‘Ajude-nos [os *Heimatlosen*] a superar nossas dificuldades atuais’. O destino vos agradecerá por isso. Gostaria de lhes dizer o que precisamos acima de tudo. Gostaria também de lhes dizer o que mais precisamos. O navio se salvou, mas não tem nada além de sua carcaça. Encontramo-nos na mesma situação [nus, só temos a vida]. Se há a possibilidade de fornecer-nos o endereço de pessoas que estariam dispostas a cuidar de uma família desabrigada, essa ajuda será particularmente bem-vinda.

Na esperança de que nosso pedido de ajuda não se dissipe com o vento e que possamos esperar provas de um novo amor ao próximo, para o qual nem os mares nem os oceanos são um obstáculo, agradecemos antecipadamente com um sincero ‘Vergelt’s Gott’ [Deus lhe pague] aos benfeitores (Ruff, 1 set. 1947).

A carta da comunidade de Wiescherhöfen é um dos exemplos de união e organização de alemães deslocados no pós-Segunda Guerra Mundial. Tratando-se de um grupo numeroso, vivendo em extrema penúria, buscaram se fortalecer no coletivo, demandando por auxílio junto a instituições humanitárias no exterior, como a Cruz Vermelha, filial Porto Alegre, com forte apelo à identidade étnica comum.

## Considerações finais

Ao término de uma guerra de destruição, como foi a Segunda Guerra Mundial, restaram escombros, mortos, feridos, e um enorme contingente populacional de deslocados e refugiados, empurrados de uma fronteira a outra, acomodados de forma precária. Na Alemanha, o rescaldo da guerra ou pós-guerra foi marcado por miséria, fome, doenças, carestia de produção, produtos, alimentos, moradia, somada às intempéries climáticas. A reconstrução material das cidades arrasadas pela guerra se estendeu por mais de meia década.

Já a reconstrução das vidas destruídas pela guerra é um processo lento e inacabado, atravessado pelo lembrar e o esquecer; memórias traumáticas; a busca pelos fios familiares perdidos e o reagrupamento familiar. O ato de reservar um tempo na miséria cotidiana, lançar mão de caneta e folha de papel, e produzir uma escrita de si epistolar, destinada a um leitor imaginário no exterior, é significativo e relevante em um período no qual predominou o silenciamento, pressionado pelo trauma. Para Benjamin (1987a, p. 114-115)

está claro que as ações da experiência estão em baixa, e isso numa geração que entre 1914 e 1918^11^ viveu uma das mais terríveis experiências da história. Talvez isso não seja tão estranho como parece. Na época, já se podia notar que os combatentes tinham voltado silenciosos do campo de batalha. Mais pobres em experiências comunicáveis, e não mais ricos. Os livros de guerra que inundaram o mercado literário nos dez anos seguintes não continham experiências transmissíveis de boca em boca. Não, o fenômeno não é estranho. Porque nunca houve experiências mais radicalmente desmoralizadas que a experiência estratégica pela guerra de trincheiras, a experiência econômica pela inflação, a experiência do corpo pela fome, a experiência moral pelos governantes. Uma geração que ainda fora à escola num bonde puxado por cavalos viu-se abandonada, sem teto, numa paisagem diferente em tudo, exceto nas nuvens, e em cujo centro, num campo de forças de correntes e explosões destruidoras, estava o frágil e minúsculo corpo humano.

As duas guerras ocuparam o mesmo palco e vitimaram jovens soldados e populações civis. O término da Primeira Guerra Mundial foi o prelúdio da Segunda, cujo poder de destruição foi amplificado.

Os anos do pós-guerra são, portanto, o tempo no qual a memória começa a tomar a forma de um paradoxo: uma lembrança que esquece, um esquecimento que lembra. Tal atitude, entretanto, pode também ser encontrada em formas diferentes, mas igualmente contraditórias, na celebração contemporânea da guerra. Assim, eu gostaria de concluir com alguns pensamentos a respeito do uso político e ritual da memória em nossos tempos.

Existe o que se chama de memória pública como um ritual de autoisenção.

Sua função é delinear uma nítida separação entre o presente e o passado, a fim de declarar ser o passado um livro fechado do qual podemos lembrar mas que não tem relevância para os acontecimentos contemporâneos. Afinal de contas, tudo fazia parte do passado. E há então a memória como escândalo, uma memória que – como William Faulkner um dia definiu – nos lembra de que ‘o passado não morreu, nem mesmo passou’. A memória como ritual de autoisenção insiste nos horrores do passado para nos dizer que ‘aquilo aconteceu, mas somos diferentes e aquilo não acontecerá outra vez’. A memória como escândalo nos adverte, com Primo Levi, de que ‘aquilo aconteceu, e por isso pode acontecer outra vez’. Na celebração contemporânea, essas duas formas de memória estão muitas vezes contidas nas mesmas fórmulas, nos mesmos gestos, nas mesmas comemorações (Portelli, 2012, p.81).

Diante do exposto, as cartas remetidas à Cruz Vermelha Brasileira, em sua filial rio-grandense, com sede em Porto Alegre, são pequenos retalhos do cotidiano de sobreviventes da guerra, deslocados ou refugiados do Leste europeu, alojados em território da Alemanha, onde vivem em condições miseráveis, que permitem compreender e tecer um panorama amplo desse contexto. Escrever, além de uma catarse pessoal, é uma possibilidade de compartilhar com um outro, desconhecido e distante, um fragmento de sua trajetória de vida, seja como denúncia, seja como sensibilização. Ao fim e ao cabo, clamam por um “pacote de amor”. No mais, os Estados nacionais continuam fomentando guerras, que reverberam em milhares de deslocados e refugiados.

## Data Availability

Não estão em repositório.

## NOTAS

1 Sobre a estrutura organizacional administrativa e as bases jurídicas de atuação da CICV, Cunha e Vieira (2016, p.46) pontuam que a “relação da Cruz Vermelha com os Estados é diferente da maior parte das outras Organizações Não Governamentais Internacionais (ONGIs). As Convenções de Genebra de 1949 e seus Protocolos de 1977, sucessores da Primeira Convenção, de 1864, concedem à Organização mandato oficial para a proteger e assistir às vítimas de conflitos armados. A maioria dos países assinou acordos que atribuem à Cruz Vermelha privilégios e imunidades normalmente concedidas apenas às organizações intergovernamentais. As Convenções de Genebra e seus Protocolos Adicionais são a essência do Direito Internacional Humanitário (DIH); um conjunto de leis que rege a conduta nos conflitos armados e busca limitar seus efeitos. Protegem especificamente as pessoas que não participam dos conflitos (civis, profissionais de saúde e de socorro) e os que não mais participam das hostilidades (soldados feridos, doentes, náufragos e prisioneiros de guerra). As quatro Convenções de Genebra e do Protocolo Adicional I conferem ao CICV um mandato específico para agir em caso de conflito armado internacional. Em particular, o CICV tem o direito de visitar os prisioneiros de guerra e interna dos civis, além de amplo direito de iniciativa”.

2 No Rio Grande do Sul, há ramificações em sete filiais municipais, a saber: São Leopoldo, Sapucaia do Sul, Caxias do Sul, Tramandaí, Bento Gonçalves, Montenegro e Santa Maria (Cruz Vermelha no Rio Grande do Sul, *online*).

3 A Cruz Vermelha “gerou tecnologia médica em muitas áreas (transporte, cirurgia, hospitais e centros de emergência, entre outros), que mais tarde teve um impacto na geração de conhecimento e aplicações médicas para grupos sociais em tempos de paz. Dessa forma, a análise da CR se insere no marco do ‘humanitarismo médico’, que atuou diante das transformações sociais e urbanas decorrentes da industrialização, bem como em conflitos e guerras de maior alcance, derivados da conquista de territórios, mercados e produtos. Dessa forma, pode-se interpretar o surgimento da filantropia médica e das agências sociais voluntárias, juntamente com o envolvimento gradual do Estado na assistência médica, na pesquisa médica e no estabelecimento de serviços sociais” (Di Liscia, Alvarez, 2019, p.154).

4 Na atualidade, os refugiados utilizam-se dos modernos meios de comunicação, especialmente o celular, as redes socais e a imprensa para se comunicar e denunciar situações de descumprimento dos direitos humanos – contudo, o teor dos relatos e dos dramas humanos, decorrentes da guerra, na sua essência, são semelhantes.

5 Após a Segunda Guerra Mundial, com a infraestrutura e a administração pública colapsadas, a Alemanha foi dividida em quatro zonas de ocupação, sendo comandadas pelos comandantes-chefes das forças armadas das “três grandes” nações vencedoras da guerra: Estados Unidos, Reino Unido e União Soviética, além da França, que foi convidada para participar da Conferência de Berlim, realizada de 17 de julho a 2 de agosto de 1945.

6 Na introdução de seu estudo sobre correspondências, Javier Granda (2016, p.7) escreve: “Essas cartas não foram escritas com o objetivo de servir como fonte de estudo ou mesmo de formar uma história de vida em si. Nesse sentido, são cartas indeliberadas e totalmente alheias a qualquer esfera literária ou acadêmica”. O termo “cartas indeliberadas” está presente na apresentação espanhola das correspondências do poeta francês Arthur Rimbaud e, na versão em português, consta como “cartas sem preocupação estilística” (Rimbaud, 2008). As cartas estudadas por Granda, as cartas escritas por Rimbaud e as cartas analisadas no presente estudo foram escritas com fins práticos objetivos e informativos, inseridas nas demandas do cotidiano dos sujeitos envolvidos.

7 Sobre o trauma, a história, o luto e a perlaboração da violência em nível psicológico e sociocultural, ver a discussão teórica de Rosa (2018).

8 Sobre as cartas-pedido e os envios de pacotes, há os trabalhos de Fernandes (2005, 2013, 2015); Frotscher (2015, 2018, 2019); Neumann e Meyrer (2021).

9 A carta tem uma certificação do prefeito local. Enumera os membros da família: Benno Eisenheim, nascido em 26.7.1906; Berta, esposa, nascida em 2.4.1914; filhos: Horst, nascido em 24.12.1930; Manfred, nascido em 26.4.1937; Sonjá, nascida em 3.6.1938; Harmut, nascido em 25.4.1941; Helga, nascida em 29.9.1943.

10 A United Nations Relief and Rehabilitation Administration (Unrra) foi uma agência internacional de ajuda humanitária que representava 44 nações, amplamente dominada pelos Estados Unidos. Fundada em 1943, tornou-se parte das Nações Unidas em 1945, e em grande parte encerrou suas operações em 1947. Já a Organização Internacional de Refugiados (OIR) foi fundada em 20 de abril de 1946, para solucionar o problema dos refugiados criado pela Segunda Guerra Mundial, entrando em operação em 1948, assumindo parte das atividades da Unrra. Em 1952, suas atividades foram interrompidas e foi substituída pela Acnur.

11 Período da Primeira Guerra Mundial, que se compara aqui ao que ocorreu entre 1939 e 1945, período da Segunda Guerra Mundial.
